# Magnetic Polymer Actuators with Self-Sensing Resistive Bending Response Based on Ternary Polymer Composites

**DOI:** 10.1021/acsaelm.3c00432

**Published:** 2023-05-30

**Authors:** Ander García Díez, Nelson Pereira, Carmen R. Tubio, Jose Luis Vilas-Vilela, Carlos M. Costa, Senentxu Lanceros-Mendez

**Affiliations:** † BCMaterials, Basque Center for Materials, Applications and Nanostructures, UPV/EHU Science Park, 48940 Leioa, Spain; ‡ Centre of Physics Universities of Minho and Porto and Laboratory of Physics for Materials and Emergent Technologies, LapMET, University of Minho, Campus de Gualtar, 4710-057 Braga, Portugal; § Departamento de Química Física, Facultad de Ciencia y Tecnología, Universidad del País Vasco (UPV/EHU), Apdo. 644, 48080 Bilbao, Spain; ∥ Ikerbasque, Basque Foundation for Science, 48009 Bilbao, Spain

**Keywords:** multifunctional composites, PVDF, magnetic actuator, resistive sensors, strain sensors

## Abstract

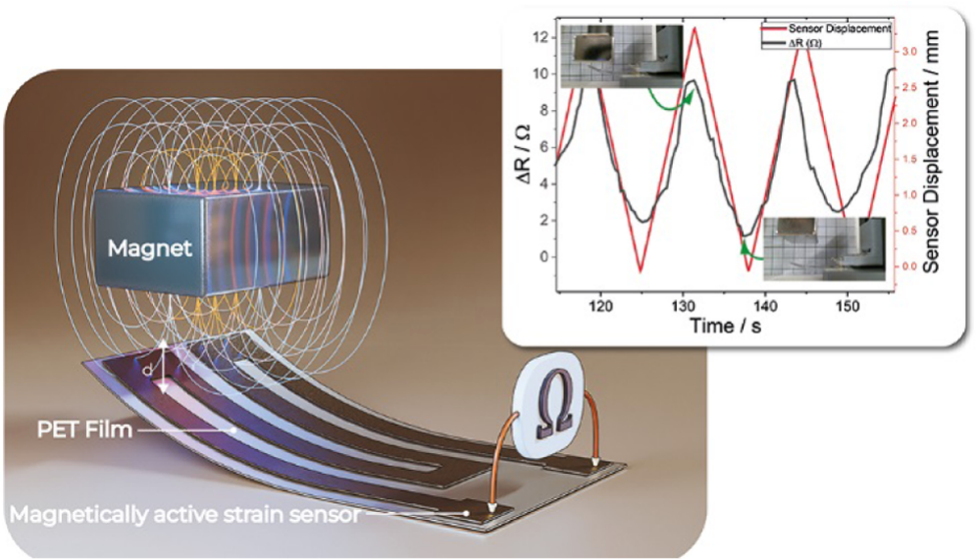

A multifunctional polymer-based composite has been designed based on poly(vinylidene fluoride) (PVDF) as polymer matrix and cobalt ferrite (CoFe_2_O_4_, CFO) and multiwalled carbon nanotubes (MWCNTs) as fillers, allowing to combine magnetic and electrical responses. The composites were prepared by solvent casting with a fixed 20 wt % concentration of CFO and varying the MWCNTs content between 0 and 3 wt %, allowing to tailor the electrical behavior. The morphology, polymer phase, and thermal and magnetic properties are nearly independent of the MWCNT filler content within the polymer matrix. On the other hand, the mechanical and electrical properties strongly depend on the MWCNT content and a maximum d.c. electrical conductivity value of 4 × 10^–4^ S·cm^–1^ has been obtained for the 20 wt %CFO-3 wt %MWCNT/PVDF sample, which is accompanied by an 11.1 emu·g^–1^ magnetization. The suitability of this composite for magnetic actuators with self-sensing strain characteristics is demonstrated with excellent response and reproducibility.

## Introduction

Current technological developments increasingly demand connectivity and interactivity, the use of smart and multifunctional materials being most suited to address those requirements. In fact, they are being applied in a wide variety of fields such as microrobotics,^1^ energy harvesting,^2^ actuators,^3^ biomedical devices,^4^ or sensors,^5^ to name a few.

Smart materials can be developed in a variety of shapes and forms also based on different material types, including polymers, ceramic, and metals.^6^ Nevertheless, the use of organic-inorganic composites is one of the most widespread approaches, due to the tunability of their properties depending on the selection of polymeric matrix (organic part) and functional fillers (inorganic), yielding a final material with enhanced or even novel properties compared to its constituents.^7^ For instance, thermoplastic polymers are commonly used as matrix material since their simple processability allows the production of complex structures.^8^ Among them, poly(vinylidene fluoride) (PVDF) and its copolymers are highlighted based on their mechanical, electrical, and chemical properties, together with their piezo-, pyro-, and ferroelectricity properties when crystallizing in specific phases.^9−11^ PVDF is a semicrystalline polymer that can crystallize into five different phases (α, β, γ, δ, and ε) depending on the processing conditions,^12^ of which the β phase is characterized by the highest electroactive response. The crystallization of the polymer in this electroactive phase can be achieved in different ways, including the introduction of specific nanoparticles,^13^ ionic liquids,^14^ carbon nanotubes,^15^ nanosheets,^16^ or by stretching^17^ and poling processes.^18^ Thus, using PVDF as the matrix for the development of polymer composites has the unique advantage of enhancement of the electroactive response when introducing fillers, aside from the properties that the filler itself adds. Owing to these enticing properties, PVDF-based composites are used for a wide range of applications.^19^

Among the many properties that can be added to polymer composites using inorganic fillers, magnetism can be highlighted, typically achieved by the introduction of magnetic nanoparticles,^20,21^ which, aside from introducing a magnetic response, also can support tailoring mechanical and dielectric properties. Another very promising set of fillers typically used for composite development are carbonaceous fillers such as carbon nanotubes (CNTs),^22^ carbon black (CB),^23^ or graphene.^24^ In general, the embedding of these carbonaceous materials significantly enhances the mechanical properties and introduces electric conductivity,^25^ two essential components for applications such as conductive patterns or piezoresistive sensors.^26^ Thus, two relevant properties nonexistent or existing in a limited number of polymers, magnetism and electrical conductivity, can be introduced into a polymer composite.

Despite the many works focusing on composites comprising a polymer matrix and one functional filler, studies that aim to introduce two functionalities on the material are still scarce. However, developing composites with two or more functional responses is extremely interesting for applications, as well as for allowing the reduction of processing steps and improving integration. Recently, some examples of these ternary composites are gaining attention with PVDF and its copolymers as polymer matrix and fillers including zeolites and ionic liquids for battery applications;^27^ Ag nanowires and TiO_2_ nanoparticles for sensing and photocatalysis;^9^ Fe_3_O_4_ nanoparticles and multiwalled carbon nanotubes (MWCNT) for embedded capacitors;^28^ graphene quantum dots and cobalt ferrite with focus on improving the dielectric response;^29^ BiFeO_3_ and CoFe_2_O_4_ for multiferroic and magnetoelectric responses;^30^ barium titanate (BT) and thermally reduced graphene oxide (TGO),^31^ as well as graphene oxide (GO) and an ionic liquid (IL), 1-vinyl-3-ethylimidazolium tetrafluoroborate ([VEIM] [BF_4_])^32^ for improving dielectric constant while maintaining low dielectric losses; CaCu_3_Ti_4_O_12_ (CCTO) functionalized by silver (Ag) nanoparticles for enhanced dielectric properties;^33^ nano-sized Ba(Fe_0.5_Nb_0.5_)O_3_ (BFN) crystallites with Ni crystallites for embedded capacitor applications;^34^ or graphene oxide (GO) and lithium chloride (LiCl) tricomposites for hazardous dye adsorption and rejection.^35^

Considering the state of the art on ternary composites based on PVDF, there is no composite based on PVDF or any other polymer with a combination of cobalt ferrite oxide (CFO) and multiwalled carbon nanotubes (MWCNTs) as fillers, which will allow the development of magnetic actuators with self-sensing resistive response. CFO and MWCNTs have excellent magnetic^36^ and electrical properties,^37^ respectively, and therefore the focus of this work is the development of a novel ternary PVDF-based nanocomposite with both magnetic response and electrical conductivity. The composites have been prepared at a fixed weight concentration of 20 wt % for the CFO, enough for suitable magnetic response,^38^ varying the MWCNTs content between 0 and 3 wt %, allowing the development of conductive composites.^39^ The quantity of nanomaterials introduced into the matrix allows obtaining a functional response without compromising the mechanical integrity of the resulting composite. The obtained ternary composites were characterized both physically and chemically, in terms of their microstructure, thermal properties, magnetic response, polymer phase content, and electrical and mechanical properties. Finally, the applicability of this ternary composite as a magnetic actuator with resistive self-sensing capabilities has been demonstrated with excellent response and reproducibility without hysteresis for industrial or soft robotics applications.

## Experimental Section

### Materials

Poly(vinylidene fluoride) PVDF (Solef 6020, Mw = 700 kg·mol^–1^) was supplied by Solvay. The solvent N,N-dimethylformamide (DMF, anhydrous, 99.8%) was purchased from Sigma-Aldrich, and the multiwalled carbon nanotubes (MWCNT, NC7000, 90% carbon purity) were obtained from Nanocyl S.A. Cobalt ferrite (CoFe_2_O_4_, CFO) spherical nanoparticles of 35–55 nm size range were purchased from Nanostructured & Amorphous Materials, Inc. All materials were used as received.

### Sample Preparation

**Figure 1:**
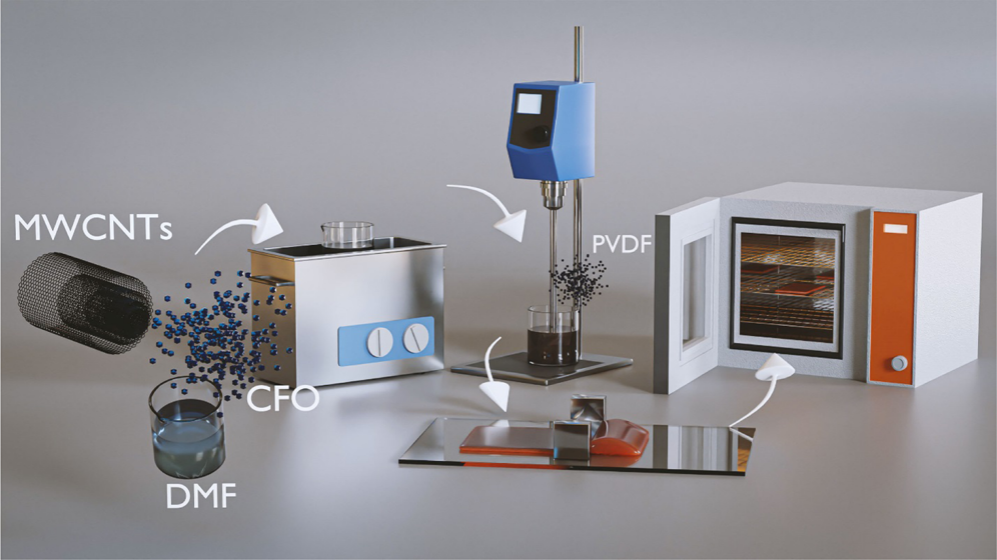
Schematic representation of the preparation procedure of the ternary composites.

PVDF-based composites were prepared with a fixed weight CFO concentration of 20 wt %, enough to achieve suitable magnetic response, without compromising mechanical characteristics.^38^ Five different samples were prepared, with varying weight fractions of MWCNTs (0, 0.5, 1, 1.5, and 3 wt %), allowing us to reach and overcome the percolation threshold.^39^ The sample processing steps are represented in Figure 1.

The appropriate amount of CFO nanoparticles and MWCNTs were mixed in 5 mL of DMF and ultrasonicated for 3 h in an ultrasound bath (ATU, model no. ATM40-3LCD). The dispersed filler solutions were then mixed with 1 g of PVDF powder and mechanically stirred for 3 h to obtain a homogeneous solution. After complete mixing, the films were prepared by doctor blade technique onto a glass substrate and subsequently heated at 210 °C for 10 min to evaporate the solvent. In addition, a neat PVDF film was fabricated following the same procedure but without the ultrasonication step, since no fillers were added in this case. Samples with an average thickness of ∼50 μm were obtained independently of the filler content.

### Samples Characterization

To evaluate the morphology and microstructure of the samples, as well as the distribution of the fillers, the composite samples were observed under a Hitachi S-4800 field emission scanning electron microscope (FE-SEM) at an acceleration voltage of 5 kV. The samples were previously coated with a 10 nm thick gold layer with an Emitech K550X sputter coater. Cryogenically fractured surfaces were also coated and scanned in the same way.

Fourier transform infrared spectroscopy (FTIR) measurements were performed at room temperature using a JASCO FT/IR-6100 spectrophotometer in the attenuated total reflection (ATR) mode in the range from 4000 to 600 cm^–1^ after 64 scans with a resolution of 4 cm^–1^.

Differential scanning calorimetry (DSC) analysis was carried out to study both the melting temperature and the degree of crystallinity of the samples, using a PerkinElmer DSC 8000 instrument under a flowing nitrogen atmosphere. Two cycles were performed, in which the temperature was increased from 25 to 200 °C, then maintained at 200 °C for 10 min, and subsequently cooled down from 200 to 25 °C, always at a rate of 10 °C·min^–1^.

The thermal stability of the samples was studied by thermogravimetric analysis (TGA) employing a TGA/SDTA 851e Mettler Toledo apparatus under a high-purity nitrogen atmosphere (99.99% minimum) at a flow rate of 10 mL·min^–1^. The samples were heated from 25 to 900 °C at 10 °C·min^–1^.

The mechanical properties of the ternary composites were evaluated in the tensile mode with an AGS-J Universal Testing Machine of Shimadzu with a load cell of 500 N and deformation of 1 mm min^–1^. To perform the measurements, the films were cut in rectangular-shaped probes of 50 mm length, 10 mm width, and ∼50 μm thick. Three specimens for each sample were measured, and the mechanical characteristics are provided as average of the three measurements. The modulus was calculated by the tangent method at 3% strain in the elastic region.

The magnetic hysteresis loops were measured at room temperature using a Microsense 2.2 Technologies vibrating sample magnetometer (VSM) from −18 to 18 kOe with a magnetization error of ±1%.

The room-temperature d.c. electrical conductivity was measured with a Keithley 487 picoammeter/voltage source by measuring the electric current with applied voltages between – 10 and 10 V for samples without MWCNT and between −0.2 and 0.2 V for samples with MWCNT. Before these measurements, the composite samples were coated on both sides with 5 mm diameter gold electrodes by sputtering (Polaron Coater SC502). The d.c. electrical conductivity (σ) was calculated using eq 1
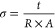
1where *t* is the thickness of the sample, *R* is the electrical resistance obtained by the slope of the *I*–*V* curves, and *A* is the area of the electrodes.

### Self-Sensing Magnetic Actuator

**Figure 2:**
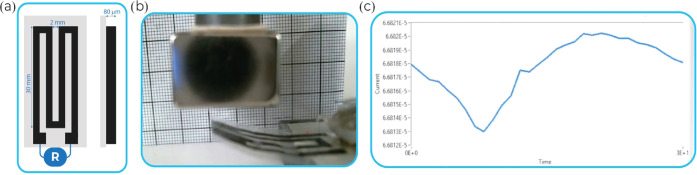
(a) Schematic representation of the actuator with dimensions. (b) Image of the approaching magnet and bending of the actuator. (c) Resistance variation under bending.

The operation principle of the magnetic actuator with resistive strain-sensing capabilities is based on the resistance variation when approached by a magnet (Figure 2). For the fabrication of the sensor, the selected composite was the PVDF/CFO 20 wt %/MWCNT 0.5 wt % one, based on the suitable combination of electrical and mechanical responses. The film was cut, with a scalpel, in a strain gauge format with a length of 126 mm, a width of 2 mm, and a thickness of 50 μm as shown in Figure 2a,b. Film tape was glued to the actuator for mechanical stability. The magnetic strain sensor was connected to a picoammeter and voltage source (Keithley 6487) with a voltage of 10 V, and the current was measured (Figure 2c).

The magnetic strain sensor was placed on a universal traction machine Shimadzu Autograph AG-IS 500N in order to use the vertical displacement control. A magnet from KJ Magnetics BX0C8-N52 (dimensions 25.4 mm × 19.05 mm × 12.7 mm, with a surface field of 5336 Gauss) was placed on the vertical axis of the sample holder, and a cyclic movement with an amplitude of 10 mm at 10, 50, and 100 mm min^–1^ (.i.e., frequencies of 0.00833, 0.041, and 0.0833 Hz, respectively) was applied above the magnetic strain sensor, as is illustrated in Figure 2b. A webcam Logitech HD 1080 was placed on the traction machine to record the video provided as Supporting Information and to measure the sensor bending displacement as a function of the approximation of the magnet.

## Results and Discussion

### Morphology

**Figure 3:**
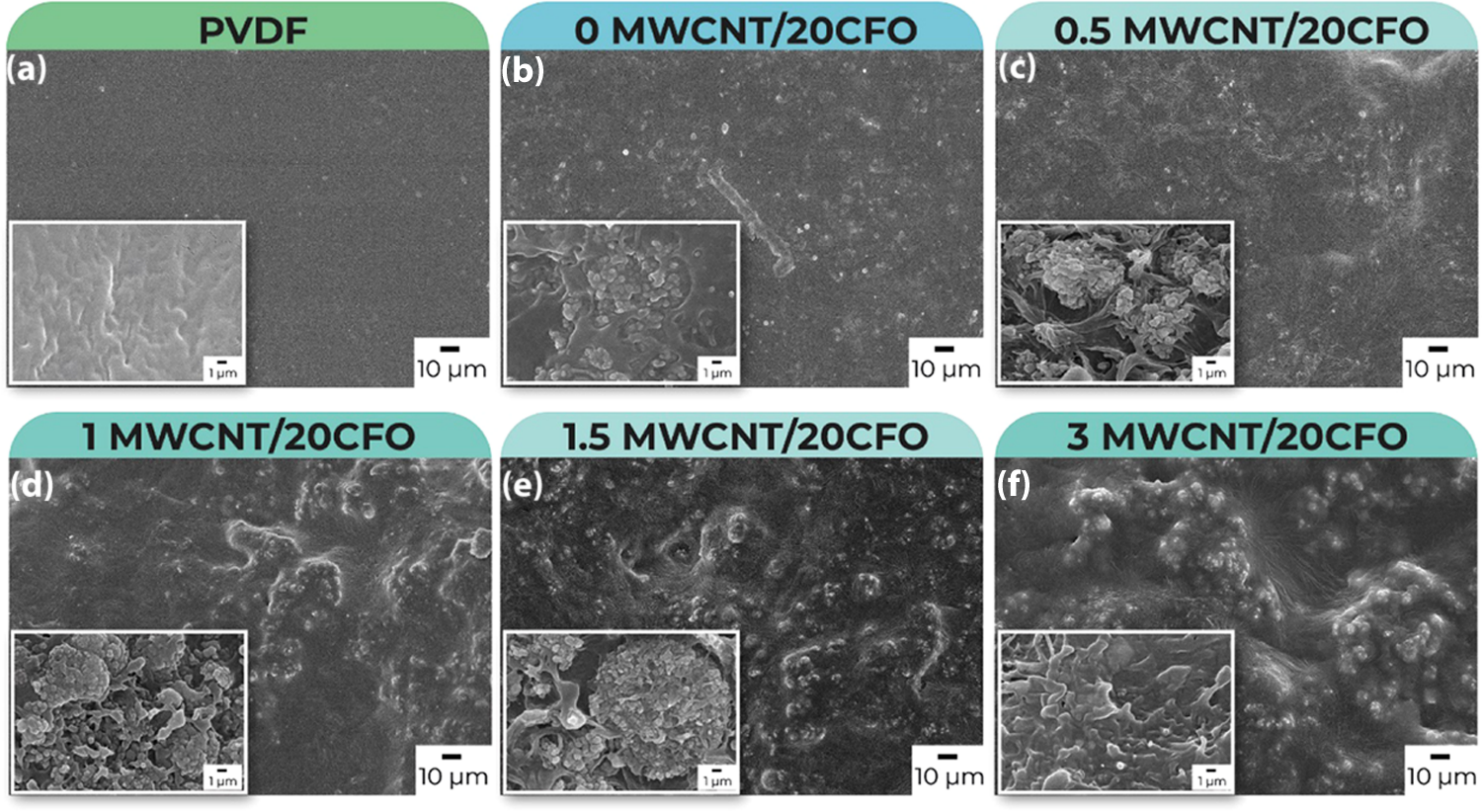
Cross-section and surface SEM images of PVDF/20CFO composites with different MWCNT contents (a–f). The scale bar represents 1 and 10 μm for the cross-section and surface images, respectively.

Figure 3 shows the cross section and top surface morphology of the composites with CFO and different MWCNT contents. For neat PVDF (Figure 3a), the structure is compact, without pores or voids, as corresponding for the selected processing conditions: solvent evaporation above the melting temperature of the polymer and subsequent cooling.^12^ The cross-section images show that the dense structure is maintained all across the film structure. With respect to the polymer composite with magnetic nanoparticles (Figure 3b), both cross and top surface images show that the CFO nanoparticles with spherical morphology are aggregated in small clusters, which in turn are homogeneously distributed within the polymeric matrix. Moreover, good compatibility of the fillers with the matrix is observed since no interfacial voids are observed.

Increasing MWCNT content (Figure 3c–f) also leads to a homogeneous distribution of the fillers all along the samples, but voids are observed in some regions of the samples, as verified in the cross-section images, due to poor compatibility between the different fillers. Nevertheless, the compatibility of each filler with the polymer matrix is good enough to guarantee mechanically robust and flexible samples. No significant differences in morphology are observed in the SEM images when the MWCNT content increases up to 3 wt %.

### Polymer Crystalline Phase, and Thermal and Mechanical Analysis

**Figure 4:**
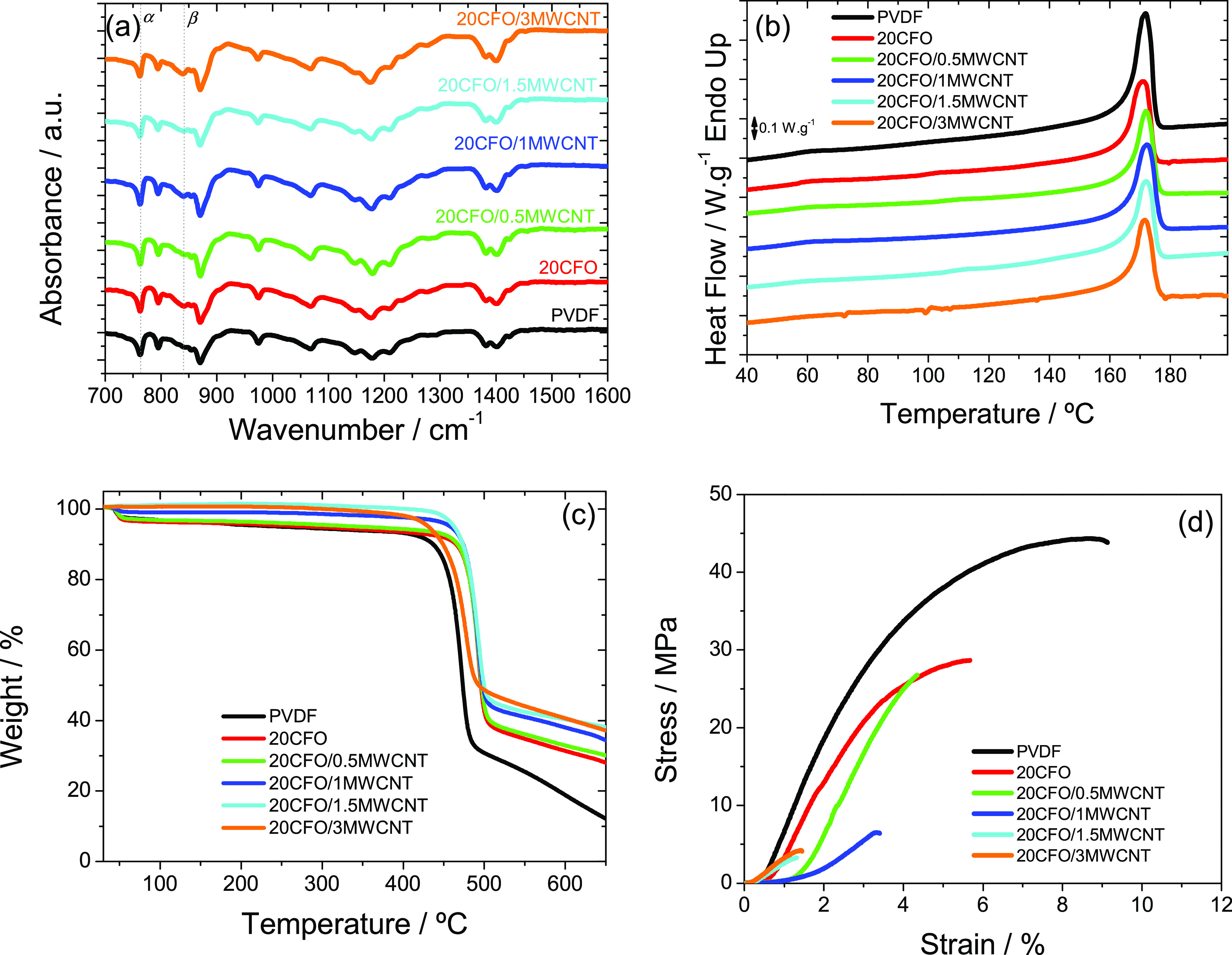
(a) ATR/FTIR spectra, (b) DSC heating scans, (c) TGA thermograms, and (d) stress–strain mechanical curves of neat PVDF and PVDF/20CFO composites with different MWCNT contents.

The effect of the addition of CFO and MWCNT in the crystalline phases of the PVDF was investigated by FTIR spectroscopy in the ATR mode, as represented in Figure 4a.

Figure 4a shows that neat dense PVDF crystallizes in the nonpolar α-phase, as confirmed by the characteristic bands associated with this phase, including the ones at 766, 795, 875, and 976 cm^–1.^^40^ At 840 cm^–1^, there is a small peak attributed to the β-phase of PVDF.^41,42^ Other distinguishable absorption bands are the ones at 1233 cm^–1^, attributed to the γ, β or γ + β-phase,^42^ and one at 1279 cm^–1^, also corresponding to the β-phase.

The spectra of the PVDF/20CFO composites with different MWCNT content are similar to the one of neat PVDF, confirming the α-phase as the main crystalline phase also in the composites. The relative β-phase content, *F*(β), has been calculated after eq 2, considering that the crystalline phase is mainly composed of α and/or β-phase crystals^43^
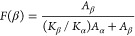
2where *A*_α_ and *A*_β_ are the absorbances at 766 and 840 cm^–1^, corresponding to bands related to the α and β-phase of the polymer, respectively, and *K*_α_ and *K*_β_ are the absorption coefficients at the same wave numbers, whose values are 6.1 × 10^–4^ and 7.7 × 10^4^ cm^2^·mol^–1^, respectively.

The fraction of β-phase in each of the samples is summarized in Table 1, confirming that the introduction of the fillers has no significant effect on the relative contents of α- and β-phases and that the processing temperature is the main factor responsible for the polymer crystalline phase,^12^ overcoming the potential nucleation effect of the different fillers. The lower β-phase content of the composite with the lower CNT content (0.5 wt %) can be attributed to the presence of the small aggregates in this sample, leading to more heterogeneous distribution of the conductive filler.

**Table 1:** Relative β-Phase Content, Melting Temperature, Percentage of Crystalline Phase, Degradation Temperature, and Young’s Modulus of the Neat PVDF Film and the Composites with CFO and Varying Contents of MWCNT

CFO/MWCNT (wt %)	β-phase (%) ± 2%	*T*_m_ (°C) ± 1 °C	*X*_C_ (%) ± 2%	*T*_deg_ (°C) ± 5 °C	*E*’/MPa	yield strength/MPa
0/0	5	172	56	442	1225 ± 103	37 ± 5
20/0	13	171	42	468	926 ± 100	25 ± 5
20/0.5	5	173	42	466	1036 ± 135	24 ± 5
20/1	10	172	49	470	337 ± 28	7 ± 3
20/1.5	11	173	39	460	314 ± 24	4 ± 2
20/3	21	172	42	439	353 ± 29	3 ± 2

Figure 4b,c shows the DSC (4b) and TGA (4c) results obtained for the different composites. In the heating DSC scan cycle (Figure 4b), the presence of an endothermic peak at around 172 °C is observed in all samples and corresponds to the melting of the crystalline phase of the polymer at *T*_m_, in agreement with the literature.^44−46^ Further, it is shown that the addition of both CFO and MWCNTs does not affect the melting temperature.

The degree of crystallinity (*X*_c_) of the samples was determined from the DSC results according to eq 3
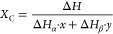
3where Δ*H* represents the melting enthalpy of the sample; Δ*H*_α_ and Δ*H*_β_ are the melting enthalpies of the α and β phases, respectively, with values of 93.07 and 103.4 J·g^–1^, respectively;^40^ and *x* and *y* correspond to the α and β phase content present in the sample. The calculated values of the degree of crystallinity are summarized in Table 1.

It is observed that the addition of the fillers, separately or combined together in the composite films, leads to a decrease of the degree of crystallinity compared to neat PVDF, as the presence of fillers induces defective crystallization of the polymer chains within the lamellae structure, the fillers acting as defects in the crystallization process of the polymer.^47^

The TGA thermograms of the different samples are depicted in Figure 4c. The thermal degradation of neat PVDF is characterized by a single degradation stage, whereas the composite films show two degradation stages, corresponding to the degradation of PVDF first and the interphase in the interface area between MWCNT nanofillers and polymer^48^ and/or the MWCNT degradation.^49^ This also leads to different residual weights in the composites.^50^ The neat PVDF sample has an onset degradation temperature (*T*_deg_) of 442 °C obtained from the established baselines in the deflection zone, and it is related to the carbon–hydrogen and carbon–fluoride scission and the formation of carbon–carbon double bond in parallel with the unzip of HF molecules from the polymer chain.^51^ Furthermore, the degradation of PVDF shifts toward slightly higher temperature values when fillers are added. Also, up to 100 °C, a mass loss stage is observed for composites with low MWCNT content probably due to residual solvent evaporation. The improvement of the onset degradation temperature with the addition of the nanofillers is noticeable, with an increase of about 48 °C for the PVDF/20CFO with 1 wt % content of MWCNTs. Regarding the samples of PVDF/20CFO with 3 wt % content of MWCNTs, a decrease of the degradation temperature due to the bigger agglomerates found in the sample with the highest filler content is observed. Thus, it is concluded that the thermal stability is improved with the addition of the nanofillers except for the PVDF/20CFO sample with 3 wt % content of MWCNTs, mainly due to the intrinsically higher thermal stability of the fillers and their good dispersion within the polymer matrix.^52^

The stress–strain mechanical curves for the different samples are shown in Figure 4d, and Table 1 presents the Young’s modulus, which was obtained for each sample considering the tangent method at 3% of elongation in the elastic region. Figure 4d shows that the typical mechanical curve of thermoplastic PVDF, characterized by an elastic and plastic region, is maintained for the composites, with the addition of both fillers decreasing the mechanical stiffness.^53^ This behavior is attributed to the accumulation of the fillers into the interphase region with the polymer.^54^Table 1 shows that the Young’s modulus of the samples decreases with respect to the one of neat PVDF, being particularly relevant for the samples with higher MWCNT contents, due to the presence of agglomerates. Furthermore, the yielding stress (Table 1) also suffers a decrease with increasing filler content due to the increased interface effects and the poor adhesion between polymer and filler, as observed in the SEM images. It is to stress that, despite those effects, all films are flexible and not fragile, with enough flexibility for magnetic actuator applications (see the Magnetic Actuator with Resistive Self-Sensing Response section).

### Magnetic and Electrical Properties

**Figure 5:**
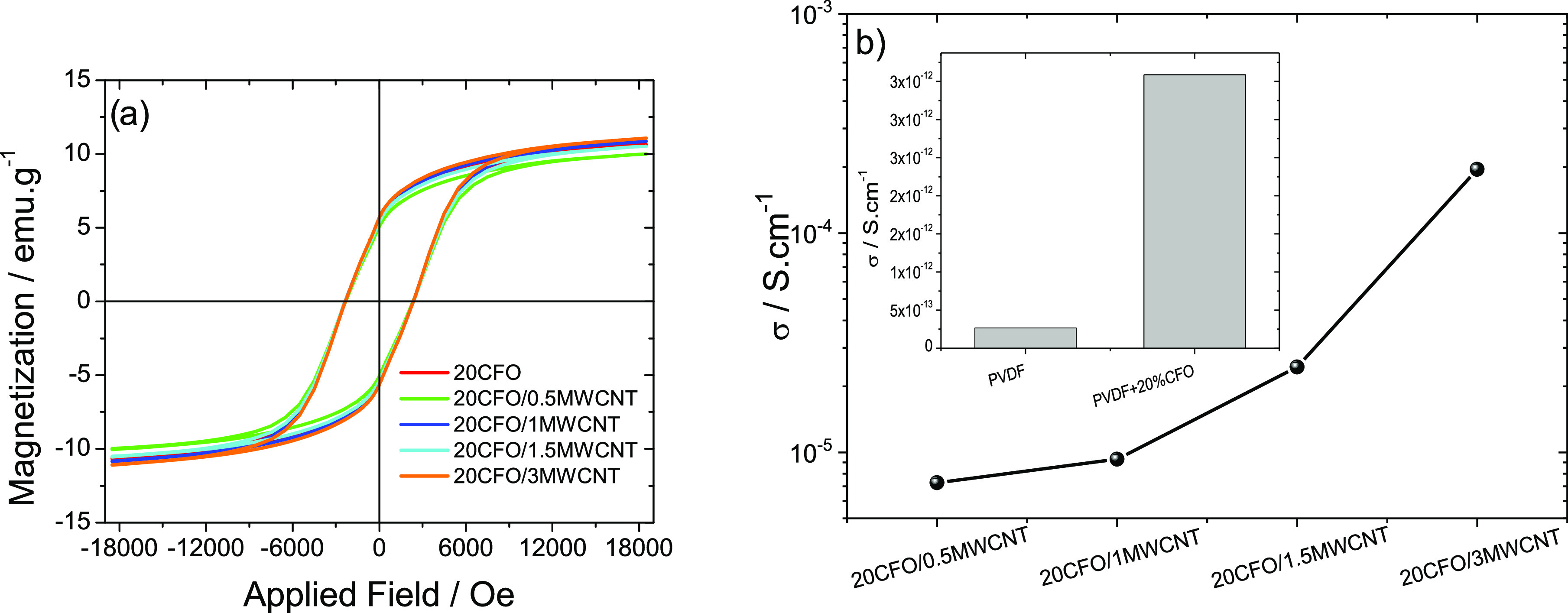
(a) Hysteresis loops at room temperature measured from −18 to 18 kOe for all composites and (b) d.c. electrical conductivity values of neat PVDF and PVDF/20CFO with different MWCNT contents.

Since CoFe_2_O_4_ nanoparticles exhibit magnetic properties,^55^ the magnetic properties of the composites were evaluated by vibration sample magnetometry. Figure 5a depicts the hysteresis loops for the PVDF/20CFO samples with different MWCNT concentrations.

These hysteresis loops indicate that the magnetic domains of the CFO nanoparticles are oriented under the influence of the magnetic field, as it is expected due to their ferromagnetic behavior.^56^ Furthermore, the bare CFO nanoparticles were measured to evaluate their saturation magnetization (*M*_S_) in order to obtain the experimental CFO concentration within the samples. The experimental concentration of the samples was calculated taking into account the measured saturation magnetization for the pure nanoparticles, which is 60 emu·g^–1^.^38^Table 2 summarizes the most relevant magnetic characteristics of the samples, including remnant magnetization (*M*_R_) and coercive field (*H*_C_).

**Table 2:** Experimental CFO Content (in Comparison with the Theoretical 20 wt %), Saturation Magnetization, Remnant Magnetization, Coercive Field, and Normalized Remanence for All of the Samples

CFO/MWCNT (wt %)	experimental CFO content (wt %)	*M*_S_ (emu·g^–1^)	*M*_R_ (emu·g^–1^)	*H*_C_ (T)	*M*_R_/*M*_S_
20/0	17	10.3	5.4	2332.4	0.52
20/0.5	17	10.3	5.3	2373.4	0.52
20/1.0	16	9.7	5.1	2353.5	0.52
20/1.5	15	8.9	4.8	2344.8	0.54
20/3.0	19	11.1	5.7	2373.8	0.51

The effective concentration of CFO nanoparticles within the polymer matrix was calculated (Table 2) considering the magnetic hysteresis loops (Figure 5a), eq 4,(57) and considering the saturation magnetization of CFO NPs (60 emu·g^–1^):^58^
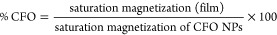
4

The differences in saturation magnetization of the samples arise from the fact that, despite all samples have been prepared with a nominal concentration of 20 wt % of CFO, some deviation from this value is obtained when considering that the manufacturing of the samples implies sonication and stirring (Figure 1), which lead to some loss of nanoparticles in the prepared ink. Despite this, the results are coherent in the sense that the higher the wt % of CFO, the higher the saturation magnetization of the films since the magnetic moment of the composite is the sum of the individual magnetic moments of the fillers.^38^

The coercive field, on the other hand, remains constant independently of the concentration of magnetic particles, which indicates that the CFO nanoparticles are well dispersed within the polymeric matrix and that they preserve their ferromagnetic behavior.^59^ It is also observed that the remanence is above half the saturation magnetization, this value corresponding to a magnetic exchange-coupled system.^60^

The slight increase in the remanent magnetization is also directly correlated to the experimental concentration of magnetic material within the samples. However, the normalized remanence (*M*_R_/*M*_S_) is constant for all of the composites, confirming that the particles are dispersed into the polymeric matrix, with no spin-canting effects, no interface reactions between the particles and the polymer,^61^ or any further reaction that can occur during the processing of the composite samples. Finally, it is worth mentioning that the introduction of carbon nanotubes has no effect on the magnetic properties of the nanocomposites, either.

Considering the electrical conductivity of the MWCNT, the d.c. electrical conductivity of the composites as a function of MWCNT content was determined from the *I*–*V* plots, the resistance values being obtained from the corresponding slope. Figure 5b shows the d.c. electrical conductivity value for all composites. The d.c. electrical conductivity of the neat polymer is 3 × 10^–13^ S·cm^–1^, and this value increases slightly for the composite with 20 wt % of CFO (4 × 10^–12^ S·cm^–1^) due to the local contribution to the electrical conductivity of the CFO nanoparticles as well as to interface effects.^62^

Figure 5b shows that the inclusion of the MWCNT in the composites leads to a strong increase of the d.c. electrical conductivity with increasing filler content, as corresponding to a percolative system,^39^ reaching a maximum value of 4 × 10^–4^ S·cm^–1^ for the 3 wt % MWCNT sample. It is to notice that for the composite with 0.5 wt % MWCNT content, the d.c. electrical conductivity is 8 × 10^–6^ S·cm^–1^, 6 orders of magnitude larger than for the polymer composite with 20 wt % CFO content, and that the further increase of the electrical conductivity is only 2 orders of magnitude by further increasing MWCNT content up to 3 wt %. Thus, a synergetic effect of the presence of both fillers is observed, and highly conductive samples are observed already with small amounts of MWCNT (0.5 wt % of MWCNT: 7.3 × 10^–6^ S·cm^–1^), despite the large amount of CFO.

### Magnetic Actuator with Resistive Self-Sensing Response

Taking into account the mechanical and electrical properties of the composites, a magnetic actuator with resistive strain self-sensing characteristics was developed with the PVDF/CFO 20 wt %/MWCNT 0.5 wt % composite based on the suitable combination of mechanical properties (Young’s modulus and flexibility) and d.c. electrical conductivity (electrical conductivity of 7.3 × 10^–6^ S·cm^–1^) (Figures 4d and 5b, respectively).

**Figure 6:**
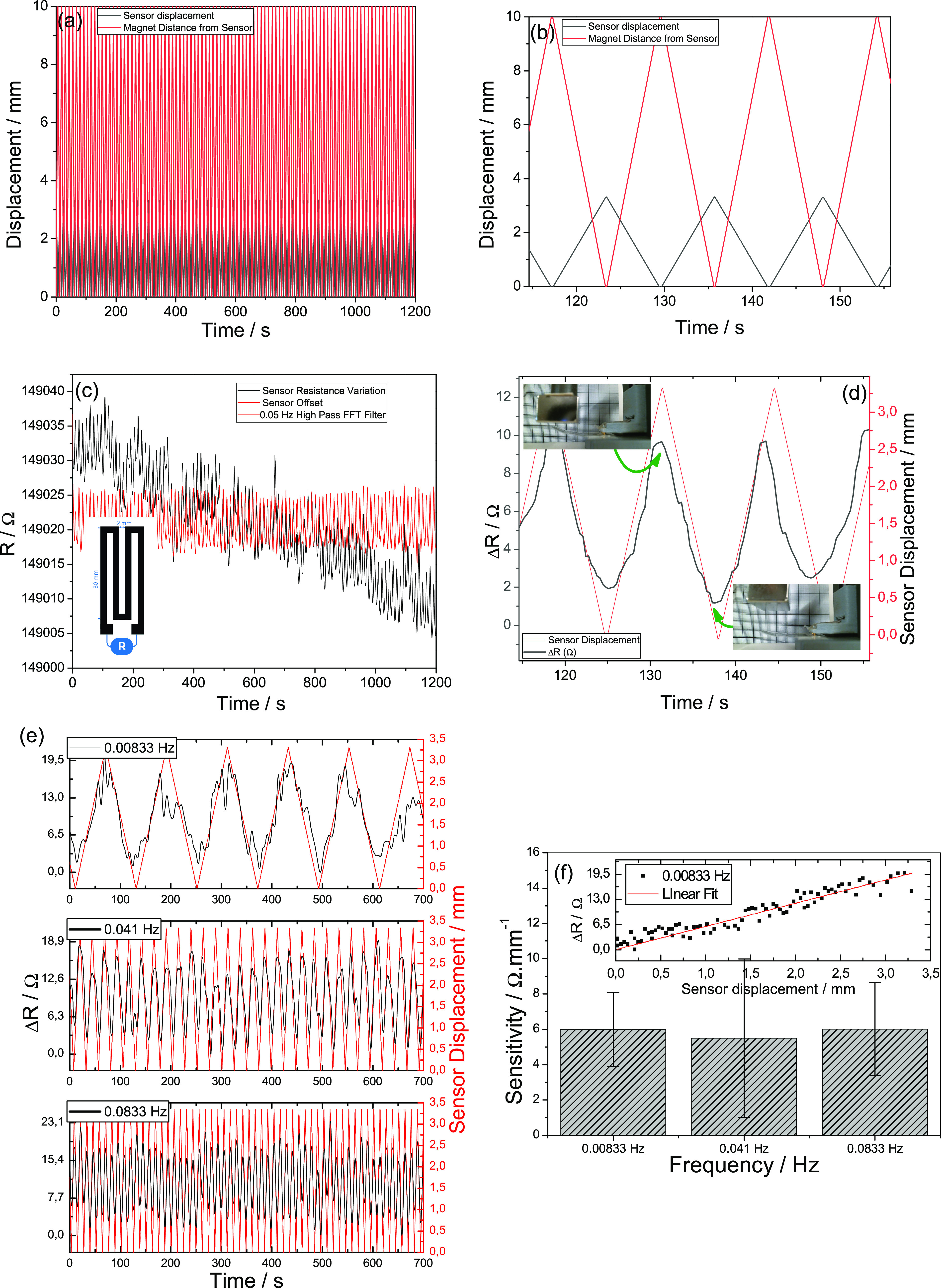
(a) 100 cycles of displacement of the magnet to the actuator and extrapolated bending deformation of the actuator based on video footage. (b) Magnification of some of the bending cycles. (c) Raw and filtered resistance variation over 100 cycles of magnetic displacement, (d) resistance variation correlation with the magnetic actuator displacement after correction of the low-frequency offset, (e) cycles performed at 0.00833, 0.041, and 0.0833 Hz, and (f) sensitivity of the sensor (insert: resistance variation as a function of the sensor displacement for determination the sensitivity value).

Figure 6a shows actuator displacement correlated to the magnet distance from the sensor for 100 cycles over time. A detailed figure is presented in Figure 6b, showing these variations for 4 cycles, demonstrating that the actuator displacement correlates with the distance to the magnet, i.e., with the magnetic field. The actuator displacement decreases with increasing distance to the magnet. The magnetic interaction between the CNT fillers and the magnet is responsible for the actuator response.

Based on the MWCNT content and the conducting properties of the composite, Figure 6c shows resistance variation of the system under magnetically induced bending after the high-pass FFT filter was applied in order to remove the DC component. Figure 6d shows an amplification of the resistance variation for 4 cycles. The magnetically induced bending^63^ leads to a strain-sensing resistance variation of approximately 12 Ω for a bending displacement of around 3 mm at the tip of the magnetic strain gauge (see video in the Supporting Information).

Figure 6e shows the cycling behavior at three different frequencies (0.00833, 0.041, and 0.0833 Hz) for a sensor displacement of approximately 3.4 mm. The sensor has a sensitivity of approximately 5.5 Ω·mm^–1^ (Figure 6f and inset) and a mean accuracy of approximately 3 Ω·mm^–1^.

For the developed actuator, the sensitivity value is high when compared to related actuator devices reported in the literature based on PVA-co-PE nanofibers with graphene oxide (GO) and silver nanowires (AgNWs) (sensitivity value ∼3 Ω·cm^–1^ and response time of 0.8 s),^64^ composites of polydimethylsiloxane (PDMS) polymer with CNT and AgNWs with a sensitivity of ∼8%/Pa,^65^ and composites based on PDMS matrix with CNT and Ti3C2Tx MXene, with a gauge factor of 11.4.^66^

Thus, the self-sensing deformation capability of the magnetic actuator, suitable for a large number of industrial applications, as well as soft robotics^67^ and biomedicine^68^ is proven.

Thus, the present work shows the development of a novel ternary composite with magnetic and electrical response suitable for magnetic actuation with self-sensing bending characteristics with excellent reproducibility.

## Conclusions

Ternary multifunctional composites with both magnetic and electrical response have been developed for advanced applications. The composites were produced by solvent casting technique based on a poly(vinylidene fluoride) (PVDF) matrix with cobalt ferrite (CoFe_2_O_4_, CFO) and multiwalled carbon nanotubes (MWCNTs) as fillers, fixing a weight concentration of 20 wt % for the CFO and varying the MWCNTs content between 0 and 3 wt %. The microstructure of these composites is compact without pores and without large aggregates. Furthermore, the filler content and type do not affect the polymer phase, the degree of crystallinity, melting and degradation temperatures, and the magnetic behavior. The mechanical properties are affected by MWCNTs filler content in the composites; Young’s modulus decreases with increasing MWCNTs.

A high d.c. electrical conductivity value of 4 × 10^–4^ S·cm^–1^ has been obtained for the 20 wt %CFO-3 wt %MWCNT/PVDF sample. The magnetic actuator capability with resistive strain-sensing characteristics has been demonstrated for the 20 wt %CFO-0.5 wt %MWCNT/PVDF sample, taking advantage of the combination of magnetic and electrical responses.

This work validates the suitability of developing multifunctional materials based on two different fillers with tailored magnetic and electrical responses for the next generation of magnetically actuator devices with self-sensing characteristics.

## Supporting Information Available

Measurement of sensor bending displacement as a function of the approximation of the magnet (MP4)
